# Supplementary material for: Molecular characterisation of the Bacillus subtilis SpbK antiphage defence system
Source: Nat Commun. 2025 Dec 29;17:1051. doi: 10.1038/s41467-025-67810-5 (PMC12847820; doi:10.1038/s41467-025-67810-5)
Supplement: Supplementary file 1 — Supplementary information [file 41467_2025_67810_MOESM1_ESM.pdf]

Supplementary Figures

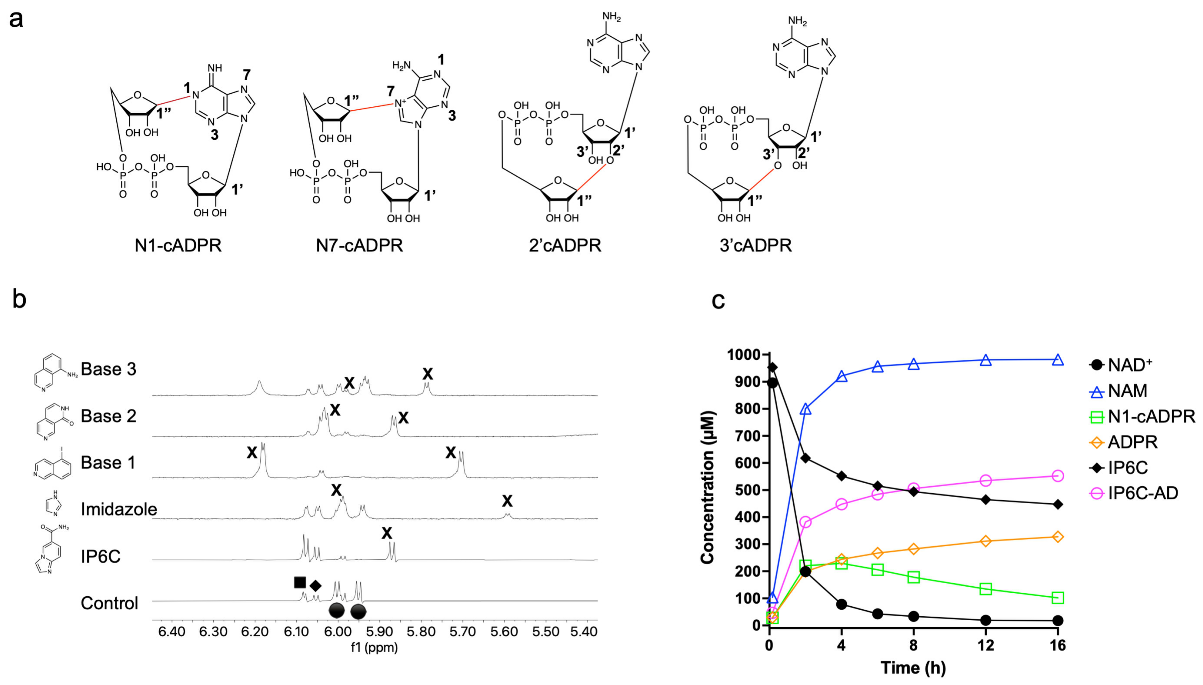

Fig. S1. Chemical structures of cADPR variants and base exchange activities of SpbK. (a) Chemical structures of N1-cADPR, N7-cADPR, 2′cADPR, and 3′cADPR, highlighting their different glycosidic linkages with 1″ anomeric position. (b) Expansions of ^{1}H NMR spectra within the anomeric region, showing base-exchange activities for SpbK in the presence of IP6C, imidazole, base **1**, **2**, and **3**. The initial NAD^+ and base concentrations were both 1 mM, while the protein concentration was 0.2 μM (control and IP6C) and 1 μM (imidazole, base **1**, **2**, and **3)**. Spectra correspond to 16 h (control and IP6C) and 22 h (imidazole, base **1**, **2**, and **3)** incubation time. Selected peaks are labelled, showing the base-exchange product (**X**), ADPR (black diamonds), and N1-cADPR (black squares) from NAD^+ (black circles). (c) Reaction progress curves for 0.2 μM SpbK + 1 mM IP6C + 1 mM NAD^+.

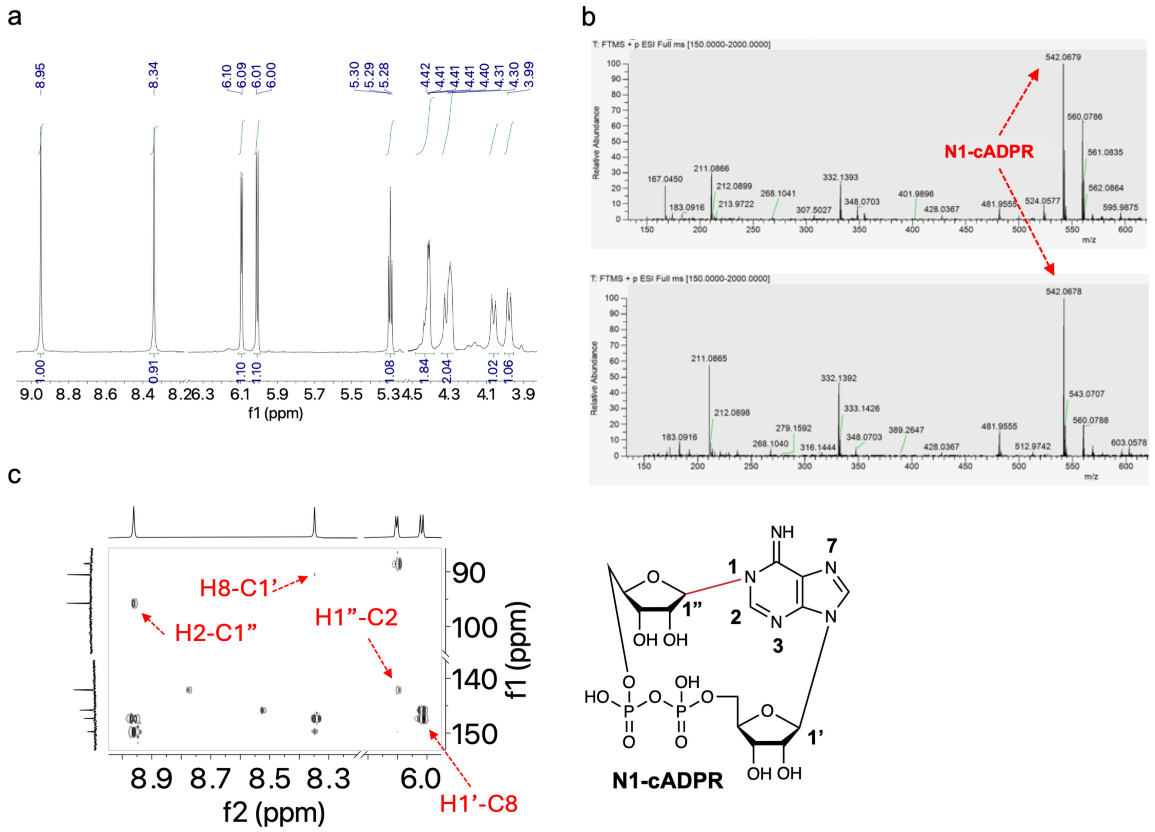

Fig. S2. MS and NMR analysis of N1-cADPR. (a) ^{1}H NMR (NOESY) analysis of purified N1-cADPR produced by SpbK. (b) MS comparison of in-house purified N1-cADPR (top) vs commercially sourced N1-cADPR (Merck C7344, bottom), both showing N1-cADPR peak at 542.068. (c) Expansions of ^{1}H-^{13}C HMBC spectra, showing correlations through glycosidic linkages for N1-cADPR. ppm, parts per million.

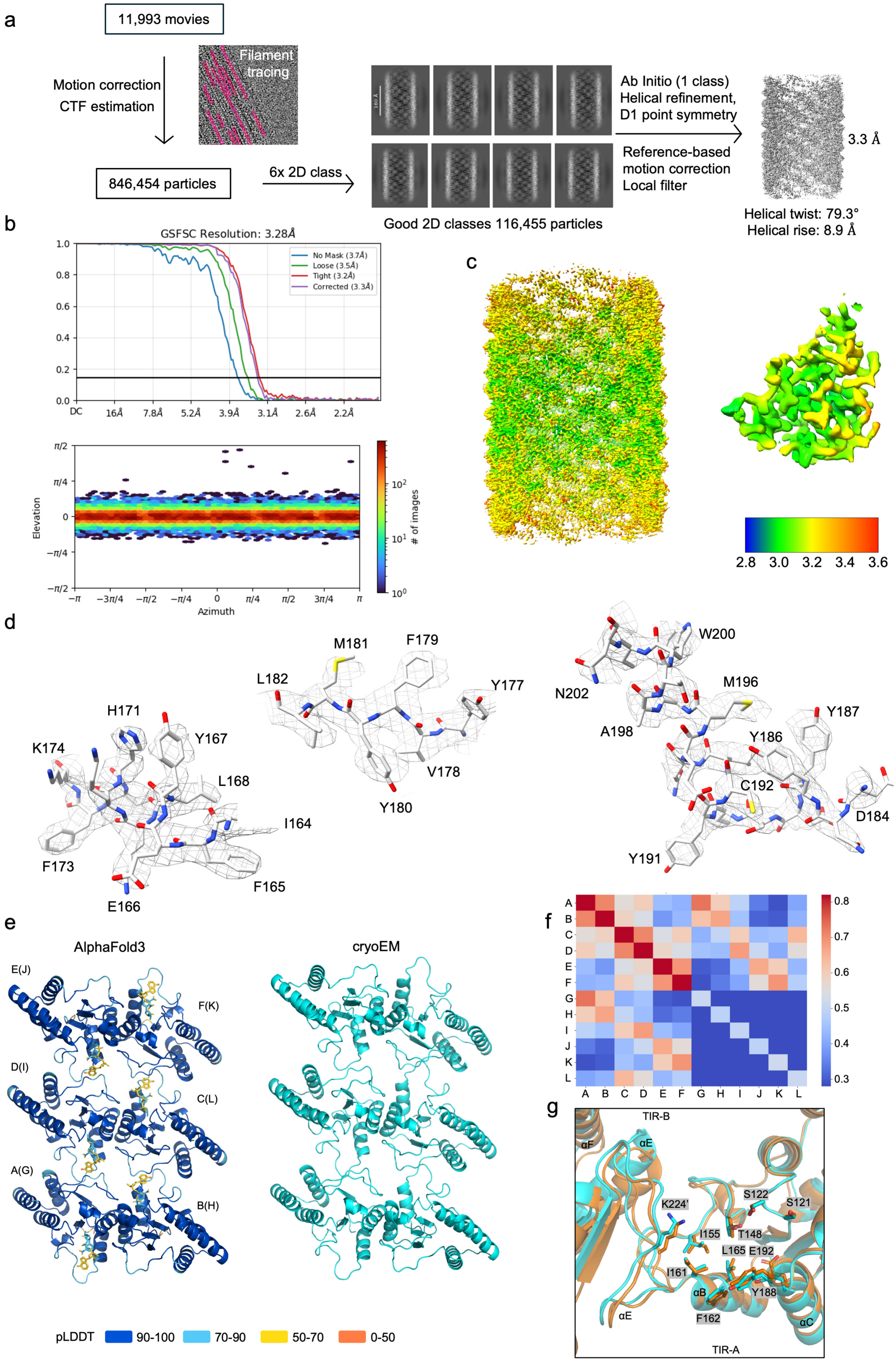

Fig. S3. Cryo-EM reconstruction of SpbK filament and AlphaFold modelling. (a) Flow-

chart of the cryo-EM processing steps. (b) Gold-standard FSC curves of the final 3D reconstruction (top) and final Euler angle distribution (bottom). (c) Local-resolution distribution of the final map. (d) Representative electrostatic potential density maps of the SpbK TIR domain. (e) Right: Cartoon representation of the SpbK protofilament AlphaFold 3 model (six subunits), coloured by the confidence metric, pLDDT. Chain identifiers are highlighted; the letter in parentheses corresponds to NAD^+. Left: CryoEM structure of SpbK protofilament (six subunits). (f) Chain ipTM heatmap of the SpbK protofilament, coloured from low score in blue, to high score in red. (g) Comparison of active site region in AlphaFold 3 model (orange) and cryoEM structure (cyan).

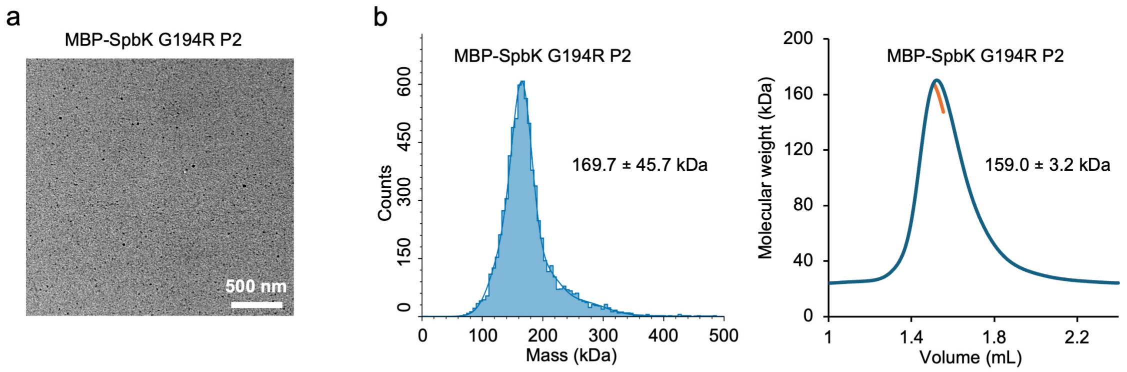

Fig. S4 Characterisation of MPB-SpbK G194R P2. (a) Representative negative-stain EM image of MBP-SpbK G194R P2 incubated with TEV protease. Micrographs were recorded from two grids from two independent samples. (b) Mass-photometry and SEC-MALS analysis of MBP-SpbK G194R P2. In the SEC-MALS analysis the blue line represents the refractive index trace, while the orange line represents the average molecular mass distribution across the peak. Mass photometry experiments were conducted three times with similar results.

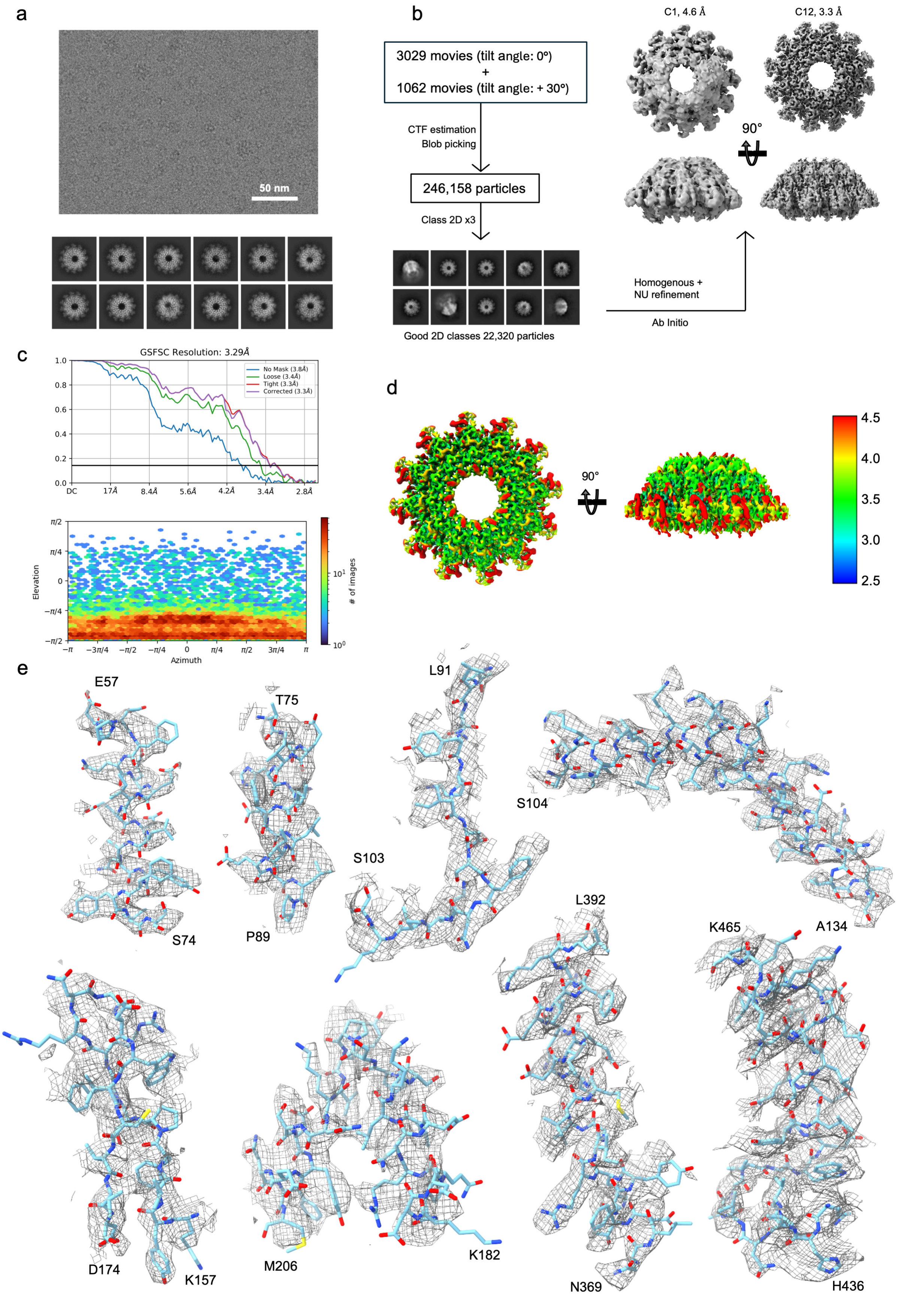

Fig. S5. CryoEM reconstruction of YonE. (a) Representative cryoEM image (top) and 2D

class averages (bottom) from untilted YonE dataset. (b) Flow-chart of the cryo-EM processing steps. (c) Gold-standard FSC curves of the final 3D reconstruction (top) and final Euler angle distribution (bottom) (d) Local-resolution distribution of the final map. (e) Representative electrostatic potential density maps.

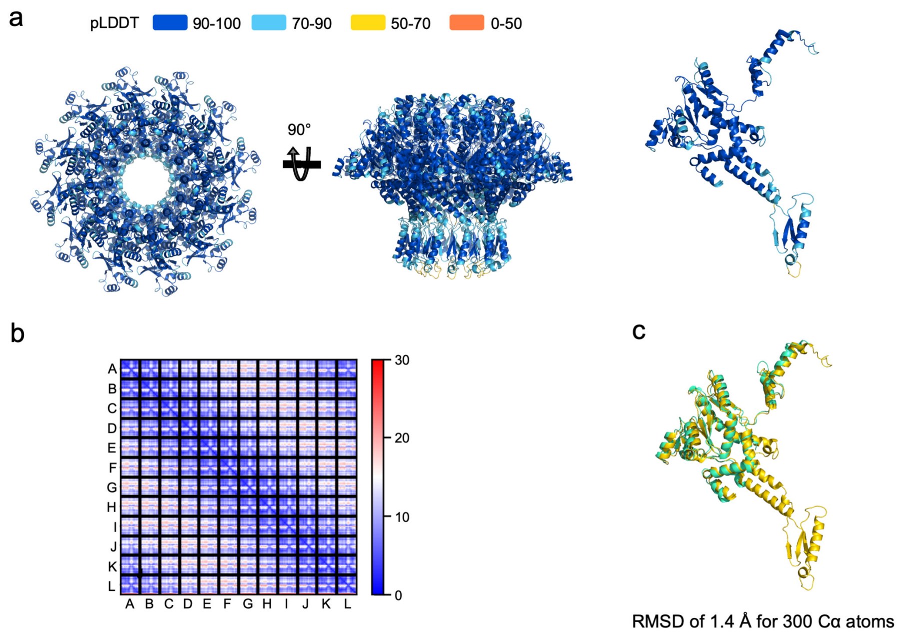

Fig. S6. AlphaFold analysis of YonE. (a) Cartoon representation of the YonE AlphaFold 2 model (top view, side view and individual monomer), coloured by the confidence metric, pLDDT. (b) Predicted aligned error (PAE) heat map for the model in (a). The map is coloured from low PAE score in blue, to high PAE score in red. (c) Structural superpositions of YonE cryo-EM structure (cyan) and AlphaFold 2 model (gold); RMSD of 1.4 Å for 300 Cα atoms.

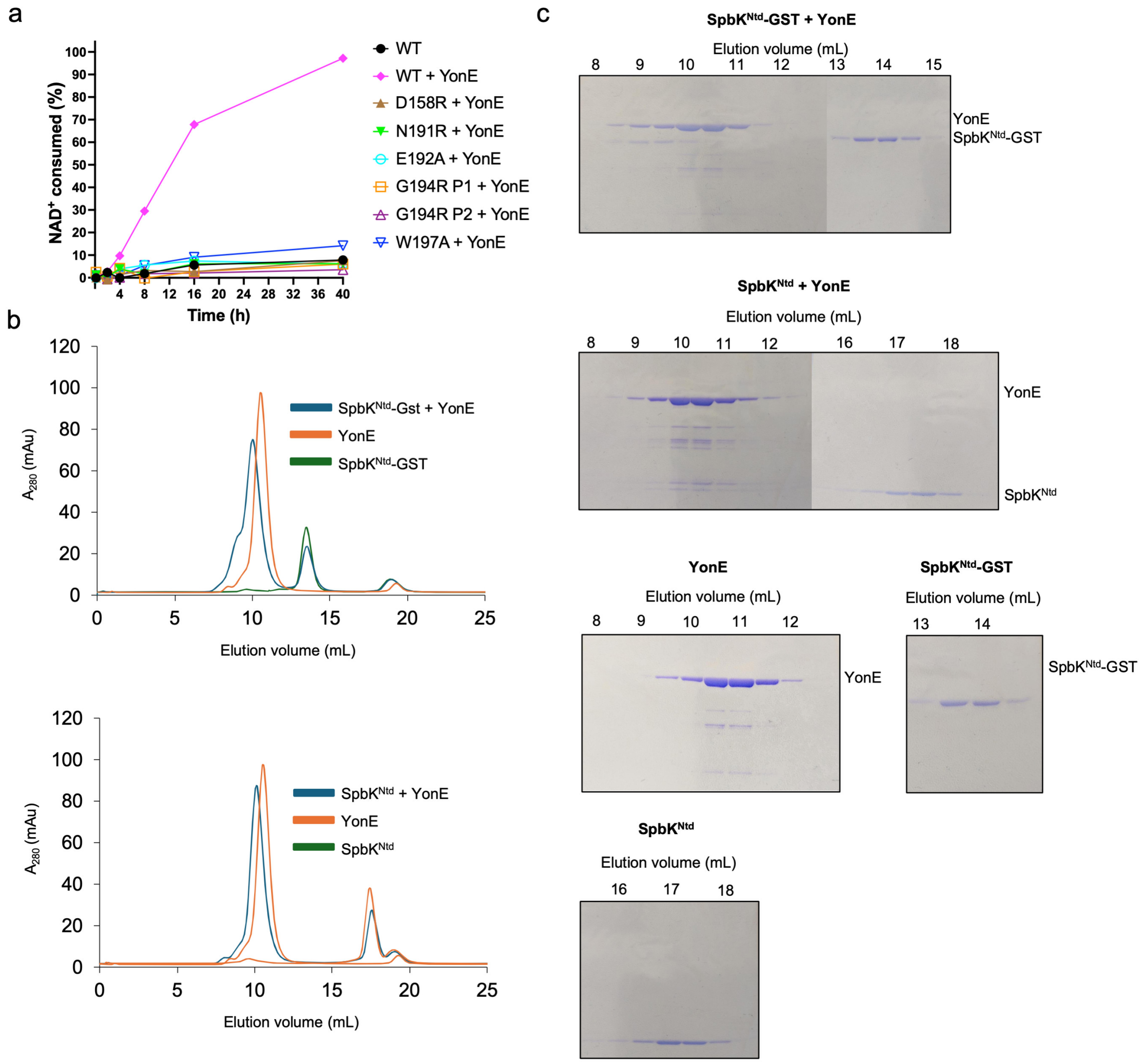

Fig. S7. NMR and gel-filtration analyses of the SpbK:YonE interaction. (a) Reaction progress curves for the MBP-SpbK D158R, N191R, E192A, G194R (P1 and P2) and W197A mutants (0.2 μM) incubated with YonE (2 μM). The initial NAD^+ concentration was 500 μM. (b) Gel filtration profiles of YonE:$SpbK^{Ntd}$-GST and YonE:$SpbK^{Ntd}$ mixtures. (c) SDS-PAGE analysis of peak fractions in (b). The gels were stained with Coomassie brilliant blue. The experiments in (a-c) were performed two times with similar results.

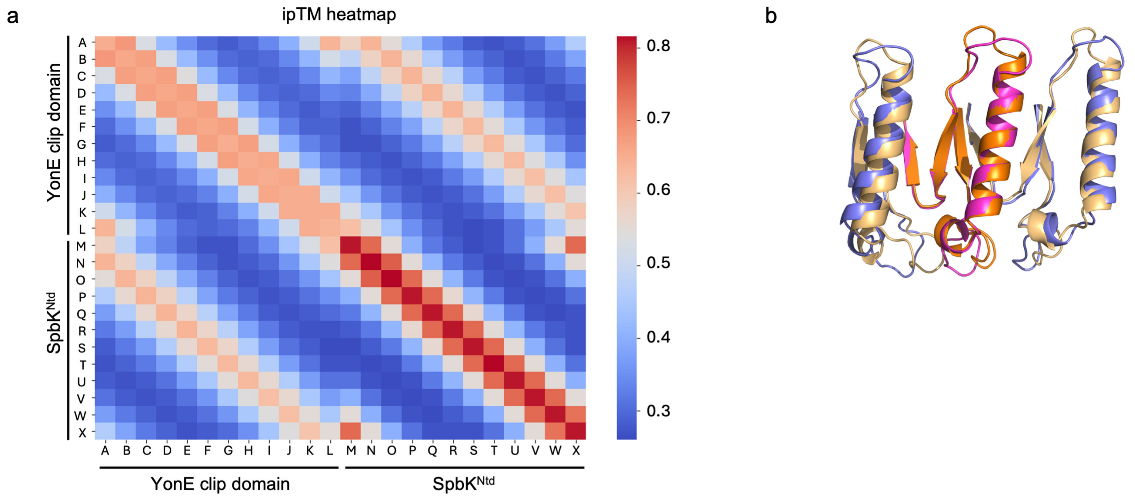

Fig. S8. AlphaFold analysis of YonE-SpbK^{Ntd} complex. (a) Chain ipTM heatmap coloured from low score in blue, to high score in red. (b) Structural superposition (Cα atoms) of YonE clip domain trimers from the dodecameric AlphaFold 2 model (magenta and slate) and the YonE:Spbk^{NTD} AlphaFold 3 model (orange and light-orange).

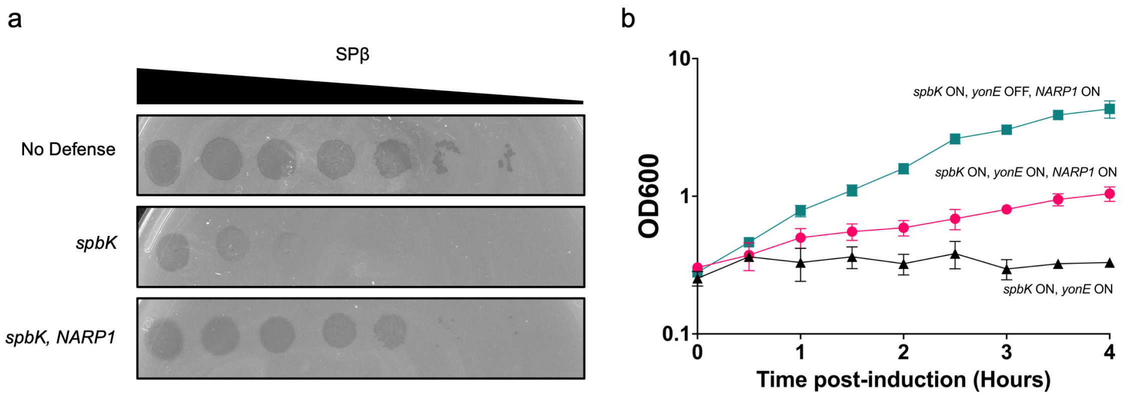

Fig. S9. Expression of NARP1 partially suppresses the growth defect caused by co-expression of *spbK and yonE*. (a) Ten-fold serial dilutions of SPβ phage were spotted onto isogenic strains expressing no anti-phage defense (CU1050, first row), *spbK* (CMJ534, middle row), or co-expressing *spbK* and *NARP1* (CLL653, bottom row). Large zones of clearing are indicative of a confluence of phage plaques and cell lysis. Small zones of clearing are indicative of individual or small clusters of phage plaques. (b) Culture turbidity by OD600 was measured and followed over time in strains co-expressing *spbK*, *yonE*, and *NARP1* (CLL656; pink circles), strains co-expressing *spbK* and *NARP1*, with uninduced *yonE* (CLL656; green squares) and strains co-expressing spbK and yonE (CMJ685; black triangles). Expression of *yonE* was induced by addition of 1 mM IPTG. Data shown in (b) are from three biological replicates. Error bars represent standard deviation.

Table S1. Assignments of N1-cADPR NMR peaks (structure shown in Fig. S2C)

Position #	^{1}H (ppm), splitting, J (Hz)	^{1}H-^{1}H COSY	^{13}C (ppm)	^{1}H-^{13}C HMBC
**1**				
**2**	8.95, s		142	C4, C6, C5, C1”
**3**				
**4**			147.5	
**5**			120	
**6**			150	
**7**				
**8**	8.34, s		146	C5, C4, C6, C1’
**9**				
**1’**	6.02, d, 6.3	H2’	90.5	C2’, C4, C8, C4’
**2’**	5.30, dd,	H1’, H3’	72.5	C1’, C4’
**3’**	4.69, br	H2’, H4’	70.5	C1’,
**4’**	4.29, br		84.8	
**5’**	4.07/3.99		64.7	
**1”**	6.10, d, 3.9		95.8	C2”, C3”, C2
**2”**	4.70 (4.71), br		88.5 (76.9)	
**3”**	4.71 (4.70), br		76.9 (88.5)	
**4”**	4.42, br		71.9	C3”, C5”
**5”**	4.43/4.33, m		64.75	C4”, C3”

Table S2. Mass photometry and MALS analysis of SpbK.

	MW (kDa) Monomer[1]	MW (kDa) Dimer[1]	MW (kDa) Mass photometry	MW (kDa) MALS
MBP-SpbK	75.6	151.2	170.0 ± 14.3	155.8 ± 0.9
$SpbK^{Ntd}$	12.4	28.8	-[2]	14.4 ± 0.4
MBP-SpbK D158R	75.7	151.4	151.4 ± 28.7	154.6 ± 1.7
MBP-SpbK N191R	75.7	151.4	86.3 ± 26.3	75.8 ± 12.7
MBP-SpbK G194R P1	75.7	151.3	93.0 ± 31.3	68.3 ± 5.3
MBP-SpbK G194R P2	75.7	151.3	169.7 ± 45.7	159.0 ± 3.2
MBP-SpbK W197A	75.6	151.1	92.7 ± 32.3	75.9 ± 4.1

[1]Calculated from amino acid sequence.

[2]$SpbK^{Ntd}$ monomeric MW is below size limit of instrument

Table S3. Cryo-EM data collection, refinement and validation statistics

	SpbK filament	YonE
**Data collection and processing**		
Microscope	JEOL CryoARM 300	JEOL CryoARM 300
Detector	Gatan K3	Gatan K3
Voltage (kV)	300	300
Nominal magnification	100,000	100,000
Pixel size (Å)	0.4864	0.4864
Defocus range (μm)	-0.5 - 2.5	-0.5 - 2.5
Total exposure (e/Å^2)	40	40
Exposure per frame (e/Å^2)	1	1
Total micrographs (no.)	11,993	4,091
Total extracted particles (no.)	846,454	246,158
Final particles (no.)	116,455	22,320
Point group symmetry	D1	C12
Helical rise (Å)	8.92	-
Helical rotation (degrees)	79.33	-
Map resolution (Å)	3.3	3.3
FSC threshold	0.143	0.143
**Refinement**		
Model resolution (Å)	3.3	3.3
FSC threshold	0.143	0.143
Map correlation coefficient		
Volume		0.71
Map sharpening B-factor (Å^2)	125.9	77.6
Model composition		
Number of chains	10	12
Non-hydrogen atoms	12800	30,144
Residues	1560	3,600
Water	0	0
Ligands	0	0
B-factors (Å^2)		
Protein	41.21	58.89
Ligand		
Bonds (RMSD)		
Length (Å) (> 4σ)	0.002	0.003
Angles (°) (> 4σ)	0.477	0.519
**Validation**		
Molprobity score	1.51	1.63
Clash-score	3.76	6.82
Ramachandran plot (%)		
Outliers		0.00
Allowed	5.00	3.72

Favored	95.00	96.28
Rotamer outliers (%)	0.07	0.24
Cß outliers (%)	0.00	0.00
Peptide plane (%)		
Cis proline/general	0.00	0.00
Twisted proline/general	0.00	0.00
CaBLAM outliers (%)	1.12	0.68

The statistics were calculated using CryoSPARC and the phenix.validation_cryoem tool.

Table S4. Proteins used in this study.

Protein	Residue range	Vector
MBP-Spbk	SpbK: 1-266	Pet28b, N-terminal His_6-tag; TEV protease cleavage site between MBP and SpbK
SpbKNTD	SpbK:1-94	pET28b, C-terminal His_6 tag
MBP-SpbK D158R	SpbK: 1-266	pET28b, N-terminal His_6-tag; TEV protease cleavage site between MBP and SpbK
MBP-SpbK N191R	SpbK: 1-266	pET28b, N-terminal His_6-tag; TEV protease cleavage site between MBP and SpbK
MBP-SpbK G194R	SpbK: 1-266	pET28b, N-terminal His_6-tag; TEV protease cleavage site between MBP and SpbK
MBP-SpbK W197A	SpbK: 1-266	pET28b, N-terminal His_6-tag; TEV protease cleavage site between MBP and SpbK
$SpbK^{Ntd}$-GST	SpbK: 1-109	pMCSG7; N-terminal His_6-tag followed by TEV protease cleavage site; GSGGS linker between $SpbK^{Ntd}$ and GST
$SpbK^{Ntd}$-$SARM^{SAM}$	SpbK: 1-109; SARM1:409-548	pMCSG7; N-terminal His_6-tag followed by TEV protease cleavage site; GSGGS linker between $SpbK^{Ntd}$ and $SARM1^{SAM}$
YonE	YonE: 46-506	pMCSG7; N-terminal His_6-tag followed by TEV protease cleavage site
YonE A291P	YonE: 46-506	pMCSG7; N-terminal His_6-tag followed by TEV protease cleavage site

Table S5. ***B. subtilis*** **strains used.**

Strain	Genotype
PY79	ICE*Bs1*^{0} SPß^{0} (cured of ICE*Bs1* and SPβ)
CU1050	ICE*Bs1*^{0} SPß^{0} (cured of ICE*Bs1* and SPβ) *metA thrC leu codY sup-3* (*trnS-lys*)
CMJ685	PY79, *lacA*::{*spbK kan*}
CLL656	PY70, *lacA*::{*spbK kan*} *cgeD*::{Ppen-*NARP1 tet*} *amyE*::{Pspank(hy)-*yonE*)
CMJ534	CU1050, *lacA*::{*spbK kan*}
CLL653	CU1050, *lacAlacA*::{*spbK kan*} *cgeD*::{Ppen-*NARP1 tet*}
CLL730	PY79, *lacAlacA*::*spbK*(*N191R*) *kan*
CLL731	PY79, *lacAlacA*::*spbK*(*D158R*) *kan*
CLL732	PY79, *lacA*::*spbK*(*G194R*) *kan*
CLL733	PY79, *lacA*::*spbK*(*W197A*) *kan*
CLL734	PY79, *lacA*::*spbK*(*N191R*) *kan*, *spbK*::Pspank(hy)-*yonE spc*
CLL735	PY79, *lacA*::*spbK*(*D158R*) *kan*, *spbK*::Pspank(hy)-*yonE spc*
CLL736	PY79, *lacA*::*spbK*(*G194R*) *kan*, *spbK*::Pspank(hy)-*yonE spc*
CLL737	PY79, *lacA*::*spbK*(*W197A*) *kan*, *spbK*::Pspank(hy)-*yonE spc*
CLL738	CU1050, *lacA*::*spbK*(*N191R*) *kan*
CLL739	CU1050, *lacA*::*spbK*(*D158R*) *kan*
CLL740	CU1050, *lacA*::*spbK*(*G194R*) *kan*
CLL741	CU1050, *lacA*::*spbK*(*W197A*) *kan*
CMJ534	CU1050, *lacA*::{*spbK kan*}
CMJ82	CU1050 (ICE*Bs1*^{0}) (SPß^{0}) *amyE*::{*spbK cat*}
