# Supplementary material for: Molecular characterisation of the Bacillus subtilis SpbK antiphage defence system
Source: Nat Commun. 2025 Dec 29;17:1051. doi: 10.1038/s41467-025-67810-5 (PMC12847820; doi:10.1038/s41467-025-67810-5)
Supplement: Supplementary file 2 — Reporting Summary [file 41467_2025_67810_MOESM2_ESM.pdf]

nature portfolio

Corresponding author(s): Thomas Ve
Yun Shi

Last updated by author(s): 30/11/25

Reporting Summary

Nature Portfolio wishes to improve the reproducibility of the work that we publish. This form provides structure for consistency and transparency in reporting. For further information on Nature Portfolio policies, see our Editorial Policies and the Editorial Policy Checklist.

Statistics

For all statistical analyses, confirm that the following items are present in the figure legend, table legend, main text, or Methods section.

n/a	Confirmed	
☐	☒	The exact sample size (n) for each experimental group/condition, given as a discrete number and unit of measurement
☐	☒	A statement on whether measurements were taken from distinct samples or whether the same sample was measured repeatedly
☒	☐	The statistical test(s) used AND whether they are one- or two-sided<br>*Only common tests should be described solely by name; describe more complex techniques in the Methods section.*
☒	☐	A description of all covariates tested
☒	☐	A description of any assumptions or corrections, such as tests of normality and adjustment for multiple comparisons
☐	☒	A full description of the statistical parameters including central tendency (e.g. means) or other basic estimates (e.g. regression coefficient) AND variation (e.g. standard deviation) or associated estimates of uncertainty (e.g. confidence intervals)
☒	☐	For null hypothesis testing, the test statistic (e.g. F, t, r) with confidence intervals, effect sizes, degrees of freedom and P value noted<br>*Give P values as exact values whenever suitable.*
☒	☐	For Bayesian analysis, information on the choice of priors and Markov chain Monte Carlo settings
☒	☐	For hierarchical and complex designs, identification of the appropriate level for tests and full reporting of outcomes
☒	☐	Estimates of effect sizes (e.g. Cohen's d, Pearson's r), indicating how they were calculated

Our web collection on statistics for biologists contains articles on many of the points above.

Software and code

Policy information about availability of computer code

Data collection	TopSpin™ 4.4.0 (Bruker), Unicorn 7 software (Cytiva), Astra 6.1 (Wyatt Technology), AcquireMP Refeyn Ltd), SerialEM v4.2.5
Data analysis	MNova 14.3.0-30573 (Mestrelab Research), DiscoverMP software, MassLynx 4.1, Astra 6.1 (Wyatt Technology), CryoSPARC v4.5.1 and v4.6.2, ModelAngelo 1.0, phenix.real_space_refine from the PHENIX suite (1.21rc1-4895), Coot 0.9.8.5, AlphaFold 2 Multimer implemented in the ColabFold interface (v1.5.1), AlphaFold 3 (https://AlphaFoldserver.com) , AlphaFold 3 local implementation (commit: 69b749c), PyMol (Schrodinger) 2.5.2, PDBePISA (https://www.ebi.ac.uk/pdbe/pisa/), Chimera 1.17.3, ChimeraX 1.8

For manuscripts utilizing custom algorithms or software that are central to the research but not yet described in published literature, software must be made available to editors and reviewers. We strongly encourage code deposition in a community repository (e.g. GitHub). See the Nature Portfolio guidelines for submitting code & software for further information.

Data

Policy information about availability of data

All manuscripts must include a data availability statement. This statement should provide the following information, where applicable:

- Accession codes, unique identifiers, or web links for publicly available datasets
- A description of any restrictions on data availability
- For clinical datasets or third party data, please ensure that the statement adheres to our policy

Data availability

The cryo-EM density maps have been deposited into the Electron Microscopy Data Bank (EMDB) under accession numbers EMD-71643 https://www.ebi.ac.uk/pdbe/entry/emdb/EMD-71643] (YonE dodecamer) and EMD-71644 https://www.ebi.ac.uk/pdbe/entry/emdb/EMD-71644] (SpbK filament). The atomic coordinates have been deposited at the Protein Data Bank (PDB) under accession numbers 9PHA [https://doi.org/10.2210/pdb9PHA/pdb] (YonE dodecamer) and 9PHB [https://doi.org/10.2210/pdb9PHB/pdb] (SpbK filament). Protein structures used for analysis in this study are available in the Protein Data Bank under accession codes 4ZJN [https://doi.org/10.2210/pdb4ZJN/pdb], 7NAK [https://doi.org/10.2210/pdb7NAK/pdb], 7UN8 [https://doi.org/10.2210/pdb7UN8/pdb], 7UXU [https://doi.org/10.2210/pdb7UXU/pdb], 8K38 [https://doi.org/10.2210/pdb8K38/pdb], 8SPO [https://doi.org/10.2210/pdb8SPO/pdb] and 9CC7 https://doi.org/10.2210/pdb9CC7/pdb]. The source data underlying Figs 1c, 3a-c, 3e, 5b-g, 6b, 7a-d, 7g and Supplementary Figs. 1c, 4b, 7a-c and 9b are provided as a Source Data File. Additional data are available from the corresponding authors upon reasonable request. Source data are provided with this paper.

Research involving human participants, their data, or biological material

Policy information about studies with human participants or human data. See also policy information about sex, gender (identity/presentation), and sexual orientation and race, ethnicity and racism.

Reporting on sex and gender	This study did not involve human participants, their data or biological material.
Reporting on race, ethnicity, or other socially relevant groupings	This study did not involve human participants, their data or biological material.
Population characteristics	This study did not involve human participants, their data or biological material.
Recruitment	This study did not involve human participants, their data or biological material.
Ethics oversight	This study did not involve human participants, their data or biological material.

Note that full information on the approval of the study protocol must also be provided in the manuscript.

Field-specific reporting

Please select the one below that is the best fit for your research. If you are not sure, read the appropriate sections before making your selection.

☒ Life sciences ☐ Behavioural & social sciences ☐ Ecological, evolutionary & environmental sciences

For a reference copy of the document with all sections, see nature.com/documents/nr-reporting-summary-flat.pdf

Life sciences study design

All studies must disclose on these points even when the disclosure is negative.

Sample size	Sample sizes are reported in figure legends and generally encompass two or more replicates.
Data exclusions	During cryoEM data processing, images with CTF outliers were removed, and particles that could not be classified into useful 3D refinements were discarded during 2D or 3D classification steps. This is standard practice in cryoEM dataset processing. No other data were excluded.
Replication	All data shown could be reproduced, and most experiments and analysis presented were the result of two or more replicates.
Randomization	Samples were not randomised as it was not applicable to the study.
Blinding	Investigators were not blinded as it was not applicable for the design of the study.

Reporting for specific materials, systems and methods

We require information from authors about some types of materials, experimental systems and methods used in many studies. Here, indicate whether each material, system or method listed is relevant to your study. If you are not sure if a list item applies to your research, read the appropriate section before selecting a response.

Materials & experimental systems

n/a	Involved in the study
☒	☐ Antibodies
☒	☐ Eukaryotic cell lines
☒	☐ Palaeontology and archaeology
☒	☐ Animals and other organisms
☒	☐ Clinical data
☒	☐ Dual use research of concern
☒	☐ Plants

Methods

n/a	Involved in the study
☒	☐ ChIP-seq
☒	☐ Flow cytometry
☒	☐ MRI-based neuroimaging

Plants

Seed stocks

Report on the source of all seed stocks or other plant material used. If applicable, state the seed stock centre and catalogue number. If plant specimens were collected from the field, describe the collection location, date and sampling procedures.

Novel plant genotypes

Describe the methods by which all novel plant genotypes were produced. This includes those generated by transgenic approaches, gene editing, chemical/radiation-based mutagenesis and hybridization. For transgenic lines, describe the transformation method, the number of independent lines analyzed and the generation upon which experiments were performed. For gene-edited lines, describe the editor used, the endogenous sequence targeted for editing, the targeting guide RNA sequence (if applicable) and how the editor was applied.

Authentication

Describe any authentication procedures for each seed stock used or novel genotype generated. Describe any experiments used to assess the effect of a mutation and, where applicable, how potential secondary effects (e.g. second site T-DNA insertions, mosiacism, off-target gene editing) were examined.
